# Production of recombinant antibody fragments in *Bacillus megaterium*

**DOI:** 10.1186/1475-2859-6-2

**Published:** 2007-01-15

**Authors:** Eva Jordan, Michael Hust, Andreas Roth, Rebekka Biedendieck, Thomas Schirrmann, Dieter Jahn, Stefan Dübel

**Affiliations:** 1 Technische Universität Braunschweig, Institut für Biochemie und Biotechnologie, Abteilung Biotechnologie, Spielmannstr. 7, 38106 Braunschweig, Germany; 2 Technische Universität Braunschweig, Institut für Mikrobiologie, Spielmannstr. 7, 38106 Braunschweig, Germany

## Abstract

**Background:**

Recombinant antibodies are essential reagents for research, diagnostics and therapy. The well established production host *Escherichia coli *relies on the secretion into the periplasmic space for antibody synthesis. Due to the outer membrane of Gram-negative bacteria, only a fraction of this material reaches the medium. Recently, the Gram-positive bacterium *Bacillus megaterium *was shown to efficiently secrete recombinant proteins into the growth medium. Here we evaluated *B. megaterium *for the recombinant production of antibody fragments.

**Results:**

The lysozyme specific single chain Fv (scFv) fragment D1.3 was succesfully produced using *B. megaterium*. The impact of culture medium composition, gene expression time and culture temperatures on the production of functional scFv protein was systematically analyzed. A production and secretion at 41°C for 24 h using TB medium was optimal for this individual scFv. Interestingly, these parameters were very different to the optimal conditions for the expression of other proteins in *B. megaterium*. Per L culture supernatant, more than 400 μg of recombinant His_6_-tagged antibody fragment were purified by one step affinity chromatography. The material produced by *B. megaterium *showed an increased specific activity compared to material produced in *E. coli*.

**Conclusion:**

High yields of functional scFv antibody fragments can be produced and secreted into the culture medium by *B. megaterium*, making this production system a reasonable alternative to *E. coli*.

## Background

Recombinant antibodies and antibody fragments are important tools for research, diagnostics [1] and therapy [2]. Further, the generation of monoclonal antibodies for proteome research, ideally against each human protein, is a massive challenge [3]. Here, phage display has evolved into a valuable method for the selection of antibody fragments for this purpose [4]. Recombinant antibody fragments can be selected from huge antibody gene libraries against any target by an *in vitro *panning procedure [5,6]. Furthermore, the panning procedure can be integrated into high throughput technologies [7]. For the production of large numbers of selected antibodies the established systems like mammalian cell culture [8], *Pichia pastoris *[9] or plants [10] are expensive, complicated and time consuming. The recombinant production of antibody fragments requires the folding and association of at least two different domains (VH and VL) and includes the formation of disulphide bonds. This results in lower yields compared to single domain enzymes. Overall, the production rate is strongly dependent on the individual sequence of the antibody. However, using appropriate bacterial signal peptides, antigen binding Fv fragments consisting of the VH and VL domain of an antibody have been produced in *E. coli *[11]. Improved yields of functional material were achieved by the linking of VH and VL by a 15 – 25 amino acid linker, resulting in the production of a single polypeptide, the scFv (single chain fragment variable) [12,13]. However, Gram-negative bacteria like *E. coli *secrete proteins mostly into the periplasm [14]. Only in rare cases can the scFv be isolated in higher amounts from the supernatant [15]. The use of a Gram-positive bacterium could facilitate the scFv production due to the lack of an outer membrane allowing direct secretion of proteins into the growth medium. The Gram-positive bacteria *Bacillus brevis *[16,17] and *Bacillus subtilis *[18,19] have already been succesfully used for the production of antibody fragments. In contrast to *B. subtilis*, *B. megaterium *does not produce alkaline proteases. Another advantage of this bacterium is the high stability of plasmids during growth [20], which allows a stable gene expression in long term cultivations and bioreactors. *B. megaterium *has been used for the production of several recombinant proteins, e.g. dextransucrase [21], glucanase [22] and *Clostridium difficile *toxin A [23]. Recently, a set of free replication vectors and genetically optimized *B. megaterium *strains for the intra- and extracellular production of affinity tagged recombinant proteins were developed. They were succesfully employed for the production and purification of dextransucrase [21], levansucrase [24,25], penicillin amidase [26] and a hydrolase [27].

Methods for large scale high cell density bioreactor cultivation were established [28,29]. Here, we evaluate *B. megaterium *for its capability to produce and secrete recombinant antibody fragments.

## Results

### Construction of the pEJBmD1.3scFv vector

**Figure 1:**
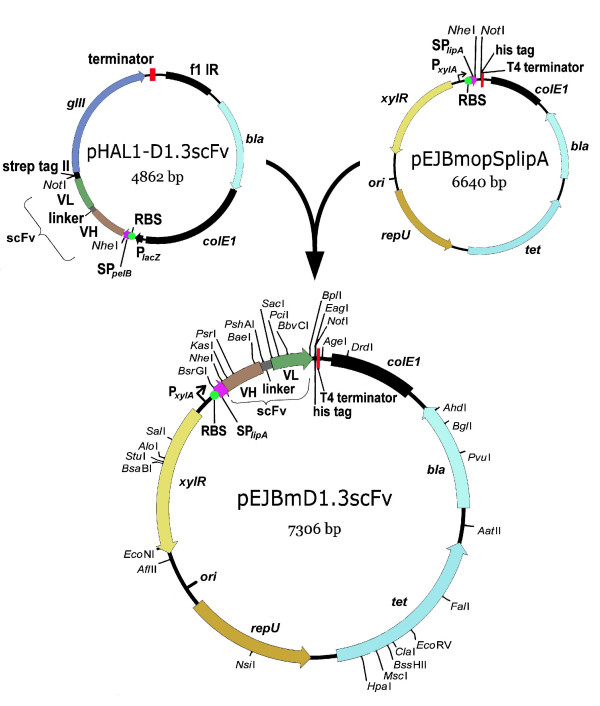
Construction of plasmid pEJBmD1.3scFv for the production of scFv antibody fragment D1.3 in *B. megaterium*. The complete scFv ORF plus the complete promoter region were verified by nucleotide sequencing. Abbreviations: *bla*: β-lactamase gene for ampicillin resistence; *colE1*: *E. coli *origin of plasmid replication; F1 IR: intergenic region of phage f1; *gIII*: fd phage gene III; His-tag: synthetic tag binding to 6xhistidine; ori: *B. megaterium *origin of plasmid replication; P_*lacZ*_: promoter of the bacterial lac operon; P_*xylA*_: xylose inducible promoter; RBS: ribosome binding site; repU: a gene essential for plasmid replication in *B. megaterium*; scFv: single chain fragment variable; SP_lipA_: signal peptide sequence of *B. megaterium *extracellular esterase LipA; SP_pelB_: signal peptide sequence of bacterial pectate lyase; *strep*-tag II: synthetic tag binding to streptactin; terminator: sequence terminating transcription; tet: tetracyclin resistence gene; VH: sequence encoding the variable fragment of the heavy chain; VL: sequence encoding the variable fragment of the light chain chain; *xylR*: xylose repressor

**Figure 2:**
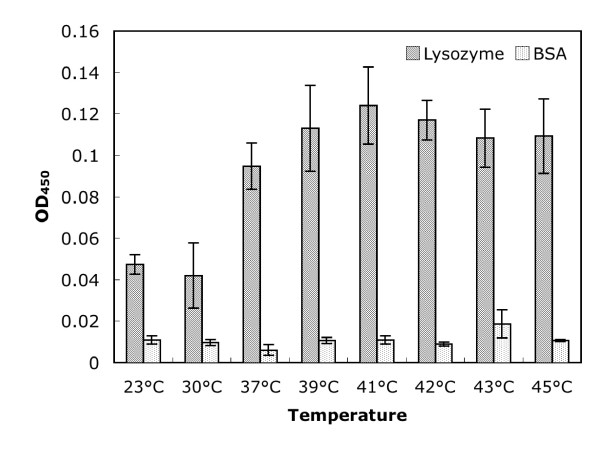
Impact of the cultivation temperature on the scFv production. Production of functional antibodies was analyzed by antigen binding ELISA of D1.3 scFvs of 50 μL culture supernatant after 24 h production in 100 mL scale. Mean values and standard deviations of data obtained from three different cultures are given. Antigens: 1 μg/well lysozyme or 1 μg/well control protein BSA. The D1.3 antibodies were detected using mAb mouse anti-His and goat anti-mouse IgGHRP (Fab specific).

The vector pEJBmD1.3scFv for the production and the export of the lysozyme specific single chain Fv (scFv) antibody fragment was constructed from the *B. megaterium *expression vector pHIS1525 [25]. To obtain pEJBmopSplipA codon usage optimized DNA encoding the signal peptide sp_lipA _of *B. megaterium *lipase A was integrated and a residual *E. coli *tetracycline gene fragment was removed from the vector. The scFv gene fragment encoding the murine anti-hen egg white lysozyme antibody D1.3 [30,31] was amplified by polymerase chain reaction (PCR) from the vector pHAL1-D1.3scFv, a pHAL1-D1.3 variant [32], and cloned into pEJBmopSplipA, resulting in the vector pEJBMD1.3scFv. The structure of the vector pEJBmD1.3scFv is given in figure 1. Efficient cloning of gene fragments encoding antibody fragments in *E. coli *DH10B was sustained by the cotransformation of pMMEc4 encoding the xylose repressor gene *xyl*R under control of an arabinose promoter. Continuous *xyl*R expression led to the succesful repression of the otherwise leaky *B. megaterium xyl*A promoter in *E. coli*. After transformation of *B. megaterium *with this vector and the induction of gene expression with xylose, antigen binding by culture supernatant was confirmed by lysozyme ELISA (experimental setup as shown in Fig. 2) (data not shown). However, the initial yields were low and required significant optimization.

### Production temperature

The impact of the temperature on the xylose induced production of scFv in *B. megaterium *was evaluated first. Eight different temperatures from 23°C to 45°C were analyzed using LB medium for cultivation and a gene expresion time of 24 h. The yields of functional scFv were estimated by antigen enzyme-linked immunosorbent assay (ELISA) on lysozyme. The best yield of functional D1.3 fragments was obtained at a cultivation temperature of 41°C (Fig. 2). Interestingly, in contrast to *E. coli *secretion systems, yields did not drop significantly at even higher temperatures than 37°C.

### Optimal medium

**Figure 3:**
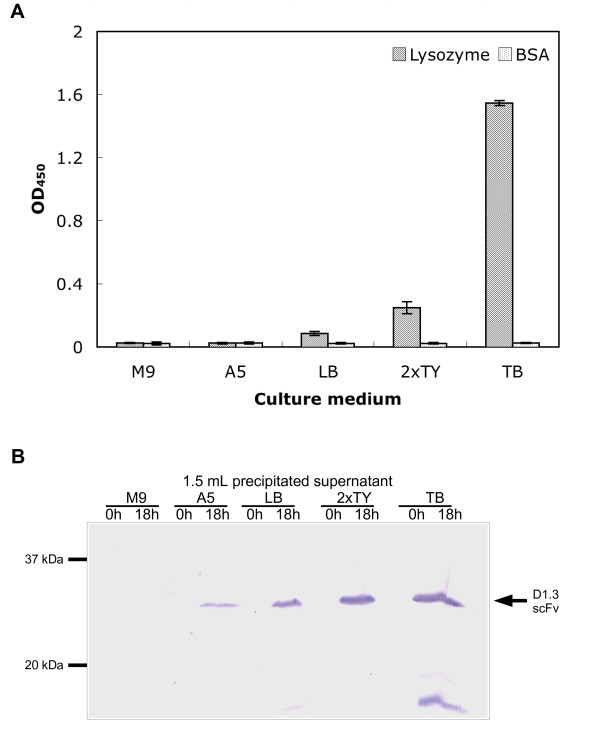
Impact of different culture media on the production of functional scFvs. **A **Antigen binding ELISA with 50 μL culture supernatant from 18 h production in 100 mL scale, performed as described in figure 2. Mean values and standard deviations of data obtained from three different cultures are given. **B **Immunoblot of culture supernatant. Ammonium sulfate precipated scFvs from 1.5 mL supernatant were separated by reducing SDS-PAGE (12 %) and detected using mAb mouse anti-His and goat anti-mouse IgG AP (Fab specific).

D1.3 scFv production at 41°C for 18 h was induced in minimal media M9 or A5, LB medium and the rich media 2xTY and TB, respectively. The secreted scFv protein was analyzed for antigen binding function by antigen ELISA (Fig. 3A) and for its molecular mass and potential degradation by SDS polyacrylamid gel electrophoresis (SDS-PAGE) and immunobloting (Fig. 3B). The minimal media did not allow production of functional scFv. The use of LB medium resulted in a low production of functional scFvs. Significant production of funtional scFvs was observed using the rich medium 2xTY. The highest production was achieved by using TB medium which is the richest of the used media. Here, the immunoblot indicated some degradation products.

### Expression time

**Figure 4:**
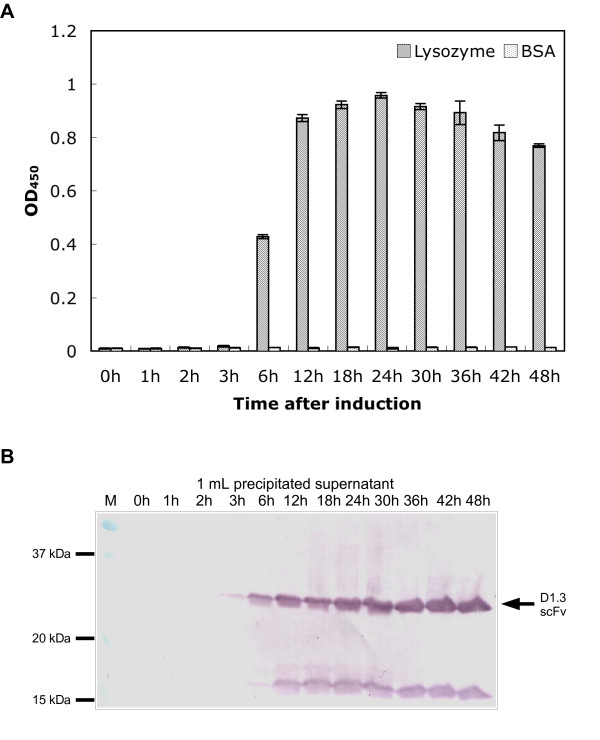
Impact of the production time on the yield of functional scFvs (TB medium, 41°C). **A **Antigen binding ELISA with 50 μL culture supernatant from different time points, performed as described in figure 2 and 3. Mean values and standard deviations of data obtained from three different cultures are given. **B **Immunoblot of produced scFvs. Ammonium sulphate precipated scFvs from 1 mL supernatant were separated by reducing SDS-PAGE (12 %) and detected as described in figure 3.

The optimal expression time for scFvs in *B. megaterium *was determined at 41°C using TB medium for activation. Samples were taken from the supernatant between 0 and 48 h after induction of the recombinant gene expression with xylose and analyzed by antigen ELISA (Fig. 4A) and immunoblot (Fig. 4B). Functional scFv fragments were detectable 6 h after induction. The amount of secreted scFv protein continuously increased until 24 hours after induction. A longer cultivation time resulted in a slight reduction of functional scFv with the total amount of antibody fragments still increasing. From 12 h on, degradation products of the antibody fragment were detected.

### Comparison of *B. megaterium *and *E. coli*

The anti-lysozyme D1.3 scFv was purified from culture supernatant of *B. megaterium *or *E. coli *periplasm and supernatant. The material was purified by immobilized metal affinity chromatography (IMAC). A yield of 410 μg/L scFv after purification was obtained from *B. megaterium*, whereas 290 μg/L were obtained from *E. coli*.

**Figure 5:**
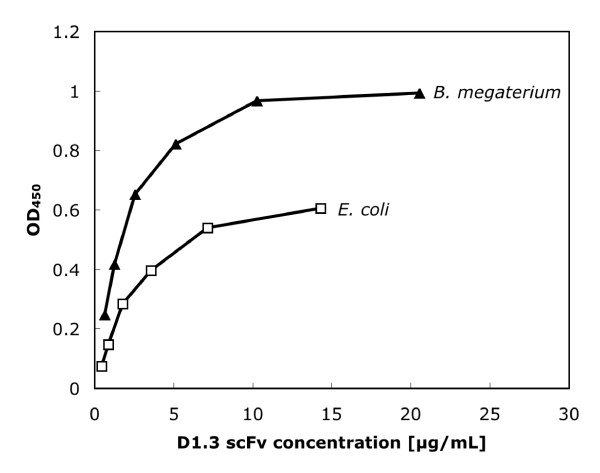
Antigen ELISA of purified D1.3 scFv from *E. coli *or *Bacillus megaterium*. Antigen: 1 μg/well lysozyme. The detection was performed as described in figure 2-4.

Serial dilutions of the purified scFv D1.3 produced by either *E. coli *or *B. megaterium *were analyzed by antigen ELISA on lysozyme (Fig. 5). The scFvs produced in *B. megaterium *showed higher specific antigen binding compared to the scFvs produced in *E. coli*, when same amounts of antibody fragments were applied to ELISA.

Both the higher concentration and the better specific activity indicate an improved enrichment from *B. megaterium *culture supernatant by a single IMAC purification step, when compared to *E. coli *material.

## Discussion

For many applications of antibodies, full length IgG material is not mandatory. For example, for research and diagnostics, correctly glycosylated IgG produced in mammalian cell lines are not necessary. Antigen binding antibody fragments, like Fab or scFv, are sufficient for many immunological standard assays e.g. immunoblot, ELISA, immunohistochemistry or immunoprecipitation. In contrast to full IgG molecules, these antigen binding fragments can conveniently be produced in *E. coli*. Although this production system is widely used, corresponding yields are somehow limited and in particular hampered by the inefficient secretion, leaving more than 90 % of the produced scFv e inside of the cell. Moreover, the majority of protein is found denatured in form of insoluble inclusion bodies [33]. These observations stimultated the search for alternative production systems.

Novel production systems could enhance the yield of functional antibody fragments or allow cheaper and easier production and purification. Various microorganism beside *E. coli*, like *B. subtilis *[18,19], *B. brevis *[16,17], *Proteus mirabilis *[34] or *Lactobacillus zeae *[35] have been used for the production of recombinant antibody fragments. Among these, Gram-positive bacteria in particular offer the opportunity to avoid the periplasm preparation step by secreting directly into the medium. In this study, the parameters for the scFv production in *B. megaterium *were established using the anti-lysozyme scFv D1.3 [30,31], an antibody fragment that has already been widely used to evaluate selection a production methods. Remarkably, the optimal yields of functional scFv fragments were obtained at 41°C, 24 h production time and in TB medium. These conditions differ significantly from those established for the production of this protein class in *E. coli, Bacillus subtilis *or *Bacillus brevis*. When using *E. coli*, a broad temperature range using 22°C, 25°C, 30°C or 37°C was successfully used for the expression of different antibody fragments with a slight advantage of lower temperatures [31,32,36-40]. For *B. subtilis *37°C, 6 – 8 h expression time and rich medium was used for the expression of scFvs [19], wheras for *Bacillus brevis *30°C, 3 days and THB medium was preferable [17]. For the production of other recombinant proteins in *B. megaterium*, like dextransucrase, 37°C was found to be optimal [21]. As it is known that folding is usually the limiting step in antibody production in various organisms [37,41,42], a possible heat shock effect, leading to improved chaperone expression, may explain these differences.

The production of the D1.3 scFv in *B. megaterium *led to a higher yield of functional antibody fragments compared to our standard *E. coli *production system, whereas a fraction of degraded scFvs were detected in the *B. megaterium *production. The production of this particular scFv can yield up to of 0.8 – 1 mg/L using a different *E. coli *expression system after extensive optimization [43]. The *B. megaterium *production strains have not been optimized compared to the long lasting optimization of *E. coli *and other *Bacillus *strains. When using *E. coli*, a yield of 1 – 2 g/L were obtained when producing a human breast tumor marker protein p185HER2 specific Fab fragment [44]. When using *Bacillus *strains, e.g. using *B. subtilis *10 – 15 mg/L of a fibrin specific scFv were produced [19] or when using *B. brevis *Fab fragments were produced at a level of 100 mg/L [16]. Furthermore, the yield is strongly depend on the individual sequence of the produced antibody fragment.

This study showed, that after a single purification step using a recombinant His_6_-tag, both the concentration of the obtained material and its functional activity are superior to *E. coli *scFv prepared in parallel.

## Conclusion

*B. megaterium *allows the production of functional scFv fragments by secretion into the culture medium. The specific activity of material obtained from *B. megaterium *was higher than that obtained from *E. coli*. Therefore, *B. megaterium *can be added to the list of microbial production hosts able to handle the demanding folding and secretion of antibody fragments. The *B. megaterium *production system offers a serious alternative to *E. coli *for the production of recombinant antibody fragments, in particular for research and diagnostic purposes.

## Methods

### Vector construction

Standard cloning procedures were performed according to Sambrook and Russell [45]. The pEJBmD1.3scFv vector was derived from the *B. megaterium *expression vector pHIS1525 [25]. The vector pHIS1525 was modified for the cloning and expression of antibody fragments. For this purpose, the terminator T4 [46] and a sequence encoding the 6xHis-tag were introduced using the two hybridised oligonucleotide primer EJoligoHisTermf and EJoligoHisTermr with the restriction sites *Sph*I and *Age*I. A residual part of the *E. coli *tetracycline resistence gene was deleted by digestion with *Afl*II and religation. Furthermore, the signal peptide sp_lipA _[25] was optimized regarding the codon usage and a *Nhe*I restriction site was introduced for further cloning steps using the overlapping oligonucleotide primer EJopSplipAf and EJopSplipAr. The signal peptide gene fragment was cloned into the *Bgl*II/*BsrG*I restriction site, resulting in the vector pEJBmopSplipA. These cloning steps were performed in *E. coli *DH5α (Invitrogen, Karlsruhe, Germany). The DNA encoding the D1.3 scFv was amplified from the plasmid pHAL1-D1.3scFv, an scFv variant of pHAL1-D1.3 [32], using the oligonucleotide primer EJD1.3NheIf and EJD1.3r and cloned into the *Nhe*I/*Not*I site to yield pEJBmD1.3scFv. This cloning step was performed in *E. coli *DH10B containing the plasmid pMMEc4, encoding the xylose repressor protein to ensure the repression of the Xylose promoter during the cloning step in *E. coli*. The plasmid pMMEc4 was derived from pBAD33 (ATCC 87402) by cloning the amplified xylose repressor gene using the oligonucleotide primer XylRfor and XylRrev from *B. megaterium *DSM319 into the *Sac*I/*Sph*I site. Transformation of *E. coli *DH10B [pMMEc4] with pEJBmD1.3scFv was performed as outlined before [45]. All affected regions of the constructs were confirmed by DNA sequencing using an ABI Prism 310 sequencer. All oligonucleotide primer sequences are given in Table 1.

**Table 1:** 

EJoligoHisTermf	5' cg ccg ctc atc acc atc acc atc act aaa aag ccc tca atg aag agg gct ttt ttt aa 3'
EJoligoHisTermr	5' ccg gtt aaa aaa agc cct ctt cat tga ggg ctt ttt agt gat ggt gat ggt gat gag cgg ccg cat g 3'
EJopSplipAf	5' cgc agt gta caa tga aaa aag tat taa tgg ctt tca tta ttt gtt tat cat taa ttt tat cag tat tag c 3'
EJopSplipAr	5' cgc aga gat ctt caa tgc ggc gct agc acc tga tgg tgg agc agc taa tac tga taa aat taa tga taa 3'
EJD1.3NheIf	5' cgc agg cta gcg ccg aag tta aac tgc agg agt cag gac ct 3'
EJD1.3r	5' cgc agg cgg ccg cct tca gct cga 3'
XylRfor	5' ata aag agc tca agg aga caa agg aat ggt tat tat tca aat tgc 3'
XylRrev	5' agg ctg cat gcc gtt cac tta act aac tta tag g 3'

### Transformation of *B. megaterium*

Transformations of non-sporulating *B. megaterium *strain MS941 [47] were performed as described by Barg et al. [48].

### Production and export of scFvs using *B. megaterium*

The D1.3 scFv was produced in shaking flasks. 100 mL medium + 10 μg/mL tetracyline were inoculated with 1 mL overnight culture at 37°C and 250 rpm. The media used were A5 pH 6.8 (30 g/L glucose, 2 g/L (NH_4_)_2_SO_4_, 0.3 g/L MgSO_4_, 0.5 g/L yeast extract, 3.5 g/L KH_2_PO_4_, 7.3 g/L NaHPO_4 _× 2H_2_O, 40 mg/L MnCl × 2H_2_O, 53 mg/L CaCl_2 _× 2H_2_O, 2.5 mg/L FeSO_4 _× 7H_2_O, 2.5 mg/L (NH_4_)_6_Mo_7_O_24 _× H_2_O, 2.5 mg/L CoCl_2 _× 6H_2_O), M9, LB, 2xTY and TB [45]. The induction was started by adding 0.5 % xylose at O.D._600 nm _= 0.3 – 0.4. The culture was shaken at 250 rpm and temperatures from 23°C to 45°C for up to 48 h. The supernatant was directly used for ELISA. For SDS-PAGE analysis and protein purification, the proteins of the supernatant were precipitated using 440 g/L ammonium sulfate.

### Production of scFvs in *E. coli*

The D1.3 scFv was produced in shaking flasks according to Dübel et al. [37] using the vector pOPE101 [49] and the *E. coli *strain XL1-Blue MRF' (Stratagene, Amsterdam, Netherland). Briefly, 300 mL 2 × TY + 100 mM glucose + 100 μg/mL ampicillin were inoculated with an overnight culture yield to O.D._600 nm _= 0.1 and cultured at 37°C and 250 rpm. The scFv production was induced by adjusting to 50 μM IPTG at O.D._600 nm _= 0.5 and shaking for 3 h at 30°C. Bacteria were harvested by 10 min at 4400 × g and 4°C. The supernatant was used for ammonium sulfate precipitation (see below). Bacteria pellets were resuspended in 30 mL ice cold PE buffer, pH 8 (20 % sucrose, 50 mM Tris, 1 mM EDTA) and incubated for 20 min on ice, interrupted by short vortexing every 2 min. Subsequently the bacteria were pelleted for 30 min at 30,000 × g and 4°C. The supernatant (periplasmic fraction) was stored at -20°C. The remaining supernatant from the first bacteria centrifugation was precipated using 132 g/300 mL ammonium sulfate, stirred 1 h at 4°C and centrifuged for 20 min at 7700 × g and 4°C. The protein pellet was dissolved in 10 mL PBS. The periplasmic fraction and the precipitated supernatant were combined and dialysed over night against PBS at 4°C.

### Immobilized metal affinity chromatography (IMAC) purification of antibody fragments

Antibody fragments were purified from *E. coli *or *B. megaterium *derived material by affinity chromatography using IMAC. Chromatography using 1 mL Chelating Sepharose Fast Flow (Amersham Biosciences, Freiburg, Germany) was performed according to the manufacturers' instruction. The protein solution was adjusted to 10 mM imidazol containing buffer (20 mM Na_2_HPO_4_, 500 mM NaCl, 10 mM imidazol) for loading The column was washed one time with 10 mM imidazol, twice with 50 mM imidazol buffer and once with PBS. Two times 50 mM NaH_2_PO_4_, 300 mM NaCl and 250 mM imidazol and finaly with phosphate buffered saline (PBS) [45], pH 7.4, 100 mM EDTA were used for the elution.

### Antigen binding ELISA

Maxisorb MTPs (Nunc, Wiesbaden, Germany) were coated with 1 μg hen egg white lysozyme or 1 μg BSA in 100 μL PBS per well overnight at 4°C. Coated wells were washed three times with PBST (PBS + 0,1% (v/v) Tween 20) and blocked with 2% (w/v) skim milk powder in PBST for 1.5 h at RT, followed by three times washing with PBST. Soluble antibody fragments were diluted in 100 μL blocking solution and incubated for 1.5 h, followed by three times washing with PBST. Soluble antibody fragments were detected with mAb mouse anti-penta His-tag (1:10000) (Qiagen, Hilden, Germany) and polyclonal goat anti-mouse IgG conjugated with horse radish peroxidase (HRP) (Fab specific) (1:10000) (Sigma, Taufkirchen, Germany) and visualised with 100 μL TMB (3,3',5,5 '-tetramethylbenzidine) substrate. The staining reaction was stopped by adding 100 μL 1 N sulphuric acid. The absorbances at 450 nm and scattered light at 620 nm were measured using a microtitre plate reader SUNRISE (Tecan, Crailsheim, Germany). The absorbance at 620 nm was subtracted.

### SDS-PAGE and Immunoblot

Soluble antibody fragments were separated by SDS-PAGE [50] and blotted onto polyvinyl fluoride (PVDF) membrane (millipore, Schwalbach, Germany). The membrane was blocked with 3% (w/v) skim milk powder in PBS for 1 h at RT. For the detection of soluble antibody fragments the mAb mouse anti-penta His-tag (1:2000) (Qiagen, Hilden, Germany) was used as first antibody and goat anti-mouse IgG (Fab specific) conjugated with alkaline phosphatase (AP) (Sigma, Taufkirchen, Germany) (1:5000) was used as second antibody. The visualisation was done by NBT/BCIP.

## Authors' contributions

EJ performed the experiments and helped to draft the manuscript. MH drafted the manuscript and participated in the design and coordination of the study. AR and RB constructed the helper plasmid pMMEc4. DJ designed and coordinated the construction of the helper plasmid and helped to draft the manuscript. TS and SD participated in the design and coordination of the study and helped to draft the manuscript. SD conceived the project and wrote the grants to fund EJ, TS and MH. All authors read and approved the final manuscript.
